# Fat-1 transgenic cattle as a model to study the function of ω-3 fatty acids

**DOI:** 10.1186/1476-511X-10-244

**Published:** 2011-12-29

**Authors:** Tao Guo, Xin F Liu, Xiang B Ding, Fei F Yang, Yong W Nie, Yu J An, Hong Guo

**Affiliations:** 1 Department of Animal Science, Tianjin Agriculture University, Tianjin 300384, China; 2 College of Animal Science, Inner Mongolia Agriculture University, Hohhot 010018, China

**Keywords:** *fat-1*, transgenic cattle, ω-3 fatty acids, gene expression, gene function

## Abstract

ω-3 polyunsaturated fatty acids have been shown to play an important role in health. Enriched with ω-3 polyunsaturated fatty acids modulate expression of a number of genes with such broad functions as cell proliferation, growth and apoptosis and cell signaling and transduction, these effects, seem to regulate coronary artery disease, hypertension, atherosclerosis, psychiatric disorders and various cancer. In this context, fat-1 transgenic cattle was designed to convert ω-6 to ω-3 fatty acids could form an ideal model to study the effect of ω-3 fatty acids on the above functions. This study focuses on the total genomic difference of gene expression between fat-1 transgenic cattle and wild-type using cDNA microarrays, several genes were found to be overexpressed or suppressed in transgenic cattle relative to wild-type, these discrepancy genes related with lipid metabolism, immunity, inflammation nervous development and fertility.

## Introduction

ω-3 fatty acids can exert a wide range of effects on cell function. In addition to being a source of energy, these fatty acids can act as determinants of the physiochemical properties of cell membranes, as substrates for the production of signaling molecules or functioning mediators, and as modulators in the regulation of gene expression. Therefore, ω-3 fatty acids can profoundly affect the physiological activity and pathological process through different mechanisms.

Mammals cannot convert ω-6 to ω-3 fatty acids automatically. Fat-1 transgenic mice showed that increased content of ω-3 fatty acids, especially ALA, EPA, DHA, in addition, the ratio of ω-6/ω-3 fatty acids is dramatically decreased in various kinds of tissues [1]. Fat-1 transgenic animal model offers an opportunity for investigating the biological functions of ω-3 fatty acids and the importance of the ratio of ω-6/ω-3 in various physiological processes and diseases. The transgenic mice was found to be normal and healthy and many generations of transgenic mouse lines have been examined and their tissue fatty acid profiles showed consistently high levels of ω-3 fatty acids, indicating that the transgene is transmittable [2]. ω-3 fatty acids have many important actions not only by themselves but also by giving raise to various biologically active compounds. ω-3 fatty acids play a significant role in various diseases and especially in cancers and neurological/psychiatric disorders [2-5].

Due to the polyunsaturated fatty acids modulated gene transcription. Considering this, we utilize the cDNA microarray that is a powerful method that allows the expression of thousands of genes to be determined simultaneously. The studies of gene expression were regulated by ω-3 fatty acids mostly on specific tissue *in vitro *or *vivo *[2,6], there are rare reports the genomic expression influenced by ω-3 fatty acids, specifically in fat-1 transgenic cattle. Here we take the fat-1 transgenic cattle as model to study the change of genomic expression influenced by the increased ω-3 fatty acids and decreased ratio of ω-6/ω-3 fatty acids in the body. Thousands of discrepancy genes generated from this experiment, we choose the representative dates to analysis and delineate the exact molecular mechanism of functions of ω-3 fatty acids.

## Materials and method

### Fat-1 transgenic cattle

Cattle were engineered to carry *fat-1 *gene from *Caenorhabditis elegan*s which can add a double bond into an unsaturated fatty acid hydrocarbon chain and convert ω-6 to ω-3 fatty acids. The transgenic cattle were provided by Inner Mongolia University, life science institute.

### RNA isolation and analysis

RNA was extracted from whole blood by TRIzol extraction protocol. To ensure the quality, total RNA was quantified by UV spectrophotometry, and the purity of total RNA was assessed by 1% agarose.

### Purification of RNA and cDNA synthesis

If the purity of total RNA was not very well, it will be influence the efficiency of probe labeling and the result of the chip hybridization. RNA was purified by using a RNeasy^® ^Mini Kit (QIAGEN, Germany), following the manufacturer's recommended protocol.

One-step of cDNA synthesis. The reaction were performed with 11.5 ul of RNA mixture (2 ug of purified RNA, 5 ul of T7 promotor primer, RNase-free Water add to 11.5 ul, then incubation for 10 min at 65°C, ice-bath for 5 min to denaturation), 4 ul of 5 × First strand buffer, 2 ul of 0.1 M DTT, 1 ul of 10 mM dNTP mix, 1 ul of MMLV RT, 0.5 ul of RNase out. The reaction condition was used lid temperature at 65°C, incubation for 2 h at 40°C, 65°C for 15 min, 4°C for 5 min.

### cRNA synthesis labeling with aaUTP and purification of cRNA

First, transcription mixture(60 ul) including 5.7 ul of RNase-free water, 20 ul of 4 × Transcription buffer, 16 ul of NTP(10 mM), 6 ul of 0.1 M DTT, 6.4 ul of 50% PEG, 4 ul of aa-UTP(25 mM), 0.5 ul of RNase OUT, 0.6 ul of Inorganic Pyrophosphatase, 0.8 ul of T7 RNA Polymerase. Afterward, 20 ul of cDNA was added into 60 ul of transcription mix and mixing. The reaction condition was used lid temperature at 60°C, incubation for 2 h at 40°C.

cRNA was purified by using a RNeasy^® ^Mini Kit(QIAGEN, Germany), following the manufacturer's recommended protocol.

### Fluorescence labeling and purification

To concentrate the 4 ug of cRNA which was above -mentioned to 6.6 ul and add 10 ul of DMSO, 3.4 ul of 0.3 M NaHCO3(pH9.0) and mixing. Cy3 was added into the 20 ul of mixture, incubation for 1 h at 25°C. Finally, 10 ul of 4 M Hydroxylamine was added and incubation for 15 min at 25°C. Fluorescence labeling cRNA also need purification, the method as same as the purification of cRNA, which was above -mentioned.

### Hybridization (4×44K microarrays)

The purified Cy3 cRNA demand to fragmentation before the hybridization, the reaction (55 ul) was performed with 875 ng of Cy3 cRNA, 11 ul of 10 × Blocking Agent, 2.2 ul of 25 × Fragmentation Buffer, Nuclease-free water added to 55 ul, incubation for 30 min at 60°C to fragmentation. 45 ul of 2 × GEx Hybridization Buffer was added into the cRNA fragmentation. 100 ul mixture was dropped onto the center of the array surface and then covered with a coverslip without any bubbles. The slides were placed into a sealed cassette to hybridize at 65°C water bath for 17 h.

After hybridization, the microarray slides were washed once with 2 × SSC, 0.1% sodium dodecyl sulfate (SDS) at 42°C for 4 min, once with 0.1 × SSC, 0.1% SDS at room temperature for 10 min and three times with 0.1 × SSC at room temperature for 1 min. The microarray slides were then washed with distilled water and spin dried. Hybridized slides were scanned at 5 μm using an Agilent chip Scanner. The scanner could scan with 100% and 10% PMT automatically, two results were combined use Agilent software automatically.

## Result and analysis

Fat-1 transgenic cattle and wild-type cattle have 43653 discrepancy expressed transcripts according to the Agilent software. It will be waste abundant time and energy to analysis all database, and some databases are meaningless to analysis, so this study we choose differentially expressed genes of p-value ≤ 0.05 and fc ≥ 1(Table 1).

**Table 1:** Gene expression that either upregulated or downregulated in the whole genome of fat-1 transgenic cattle (p-value < 0.05 and fc ≥ 1)

Genbank Accession	Gene name	Fold change
**Metabolism**		
NM_177494	carnitine palmitoyltransferase 1	1.635675
NM_174530	cytochrome P450, family 2, subfamily E, polypeptide 1	3.129168
NM_001100366	cytochrome P450, family 2, subfamily S, polypeptide 1	1.085825
NM_001099367	cytochrome P450, family 3, subfamily A, polypeptide 4	1.0726473
NM_001046391	cytochrome P450, family 4, subfamily F, polypeptide 3	1.021228
NM_174810	ATPase, H+ transporting, lysosomal 31 kDa, V1 subunit E1	1.0310035
NM_174717	ATP synthase, H+ transporting, mitochondrial Fo complex, subunit F6	1.0874296
NM_001083636	peroxisome proliferator activated receptor	1.1880234
AB257751	low density lipoprotein receptor-related protein 5	-1.1805074
NM_001077843	low density lipoprotein receptor-related protein 4	-1.5911577
**Immunity**		
XM_001250583	Indoleamine 2, 3-dioxygenase	2.0460057
XR_042605	granulocyte-macrophage colony-stimulating-factor receptor α	2.167638
NM_174358	interleukin-2 receptor α	-2.3078954
NM_174093	interleukin-1, beta	-2.8775382
NM_174086	interferon-γ	-2.1359362
NM_173923	nterleukin-6	-1.8120259
XM_591164	interleukin-10 receptor α	-1.107485
XM_615064	CD4 molecule	-1.19058
XM_001787801	WC1	-6.185475
XM_593126	lymphocyte-activation gene 3	-2.201507
	similar to Zeta-chain associated protein kinase 70 kDa	-2.379626
NM_177493	acetylserotonin O-methyltransferase	-2.1411839
NM_174589	prostaglandin E receptor 4	-1.1957332
NM_001166554	prostaglandin E synthase 2	-1.0895984
NM_001078151	mature T-cell proliferation 1	-1.0484107
BC142016	T-cell receptor delta chain	-2.3310187
XM_603087	T-cell acute lymphocytic leukemia 2	-1.1402003
NM_001075374	lymphocyte-specific protein 1	-1.2811403
NM_001102073	immunoglobulin-like domain containing receptor 2	-1.6142586
NM_001076844	lymphocyte cytosolic protein 2	-1.2404228
NM_001034720	lymphocyte cytosolic protein 1	-1.0534877
**Inflammation and cancer**		
NM_001101158	cell adhesion molecule 1	10.783385
NM_001035468	acireductone dioxygenase 1	2.893599
NM_001083481	suppression of tumorigenicity 7 like	1.1566072
NM_001035287	serpin peptidase inhibitor	7.2662635
NM_001083645	RAS-like, family 10, member A	2.4159741
NM_001101092	serine/threonine kinase 38 like	1.0883793
XM_608304	NLR family, pyrin domain containing 13	2.8275476
NM_174532	DnaJ (Hsp40) homolog, subfamily B, member 6	1.0343608
NM_175804	nuclear receptor subfamily 2, group F, member 1	1.1831405
XM_613126	chondroitin sulfate proteoglycan 4	-2.4661286
NM_001024521	TNF receptor-associated factor 7	-1.182513
XM_594145	L1 cell adhesion molecule	-2.0497224
XM_604945	adenomatosis polyposis coli 2	-2.1012108
XM_608123	laminin, alpha 4	-2.7227702
AB043995	matrix metallopeptidase 3	-2.013966
XM_597651	matrix metallopeptidase 15	-1.0679191
NM_174112	matrix metallopeptidase 1	-1.0266808
XM_604345	matrix metallopeptidase 16	-1.1511999
XM_609577	matrix metallopeptidase 20	-1.0834453
NM_001075502	nitric oxide synthase interacting protein	-1.027486
NM_001076799	nitric oxide synthase 2	-1.1507416
NM_174589	prostaglandin E receptor 4	-1.1957332
NM_174443	prostaglandin E synthase	-1.0576057
NM_001166554	prostaglandin E synthase 2	-1.0895984
DV775423	claudin 10	-1.221211
XM_601963	β-catenin	-2.1087096
XM_609364	NF-kB	-1.7619956
NM_001102498	NF-kB activating protein-like	-1.2362162
XM_582283	Huntingtin interacting protein-1	2.2835305
NM_001159566	transforming growth factor, beta receptor II	-1.1494738
NM_001035313	transforming growth factor beta 1 induced transcript 1	-1.560125
XM_001253071	transforming growth factor, beta receptor III	-1.0382366
NM_001101910	tumor protein p53 binding protein 1	-1.1203252
NM_174201	tumor protein p53	-1.1353312
NM_001076401	gamma-glutamyltransferase 7	-2.7013438
**Nervous development**		
XM_588574	protocadherin gamma subfamily A, 6	4.1054792
XM_001254336	protocadherin gamma subfamily A, 8	3.6014705
NM_001102513	protocadherin gamma subfamily B, 4	1.5915743
XM_870459	protocadherin gamma subfamily A, 9	3.789133
BC103033	potassium channel, subfamily K, member 10	1.2212783
XM_001253926	Olfactory receptor 13H1	4.0936475
NM_001076371	SEPTIN5	2.3829544
XM_608747	nucleoredoxin-like 2	6.2915673
XM_001788280	semaphorin 5B	-3.1176894
**Fertility**		
NM_001034205	Calmegin	2.228811
XM_608786	SRY (sex determining region Y)-box 8	1.8772229
NM_001076057	EF-hand calcium binding domain 6	-2.55162

In our study fat-1 transgenic cattle convert ω-6 fatty acids into ω-3 fatty acids and decrease the ratio of ω-6/ω-3 fatty acids (dates not shown), the change composition of polyunsaturated fatty acids can effects on gene expression, some genes are up regulation and some genes are down regulation, and then affect the physiological activity and pathological process through different mechanisms.

### ω-3 fatty acids on lipid metabolism

Fat-1 transgenic cattle enriched ω-3 fatty acids, ω-3 fatty acids play a major role in the regulation of several genes involved in fatty acid metabolism. There had been reported that the inﬂuenced by ω-3 fatty acids on lipolytic and lipogenic gene expression [7-9]. Hyperlipidemia is often associated with insulin resistance, coronary artery disease, hypertension [3-5,10]. Decreased ω-6/ω-3 ratio in the fat-1 mouse can enhance glucose tolerance, independent of changes in mitochondrial content [11]. Decreased in both mitochondrial content and intrinsic ability of mitochondrial to oxidize fatty acids, can contribute to lipid accumulation and development of insulin resistance [12,13], overexpression of carnitine palmitoyltransferase (CPT-1) and peroxisome proliferator activated receptor γ (PPAR-γ) increasing fatty acids oxidation and improving insulin sensitivity [14,15]. In our study, the expression of CPT-1 and PPAR-γ were up-regulation. It is important that the CYP (including CYP2E1, CYP2S1, CYP3A4, CYP4F3) encodes a member of the cytochrome P450 superfamily of enzymes that involved in the polyunsaturated fatty acids oxidation were upregulated.

To decrease the content of very low-density lipoprotein (VLDL) is benefit to coronary artery disease, hypertension. ω-3 fatty acids suppress triglyceride synthesis, VLDL secretion, and serum triglycerides [4,16]. Decrease the VLDL level can through two mechanisms involved in ω-3 fatty acid specific control of VLDL synthesis. First, decrease the VLDL expression directly, such as suppress the expression of low density lipoprotein receptor in transgenic cattle. Second, suppression of ApoCIII transcription, PPAR competes with HNF4 for binding the ApoCIII promoter. PPAR expression was increased in transgenic cattle [17,18].

ω-3 fatty acids protect against insulin resistance, coronary heart disease, hypertension by lowering triglyceride explained by the inhibition of hepatic lipogenesis and the simultaneous stimulation of mitochondrial fatty acid oxidation.

### ω-3 fatty acids on Immunity

ω-3 fatty acids has beneﬁcial effects on immune function [19]. ω-3 fatty acids regulate the immuniy through suppress the T-lymphocyte proliferation. T-lymphocyte proliferation has been shown to be inhibited in vitro by an increased concentration of free fatty acids via an eicosanoid-independent mechanism [20]. The T-lymphocyte regulates an immune response by responding to antigen, then produce cytokines. There are three major subsets of T-lymphocytes, Th (helper T cells), Tc (cytotoxic T cells), Treg (regulatory T cells). Th and Tc express the CD4 and CD8 receptors respectively. The CD4^+ ^T-lymphocyte can further be classified as either a Th-1 or Th-2 type cell depending on the types of cytokines it produces. The Th-1 type produces primarily interleukin-2 (IL-2) and interferon-γ (IFN-γ) which upregulates cell mediated immunity. The Th-2 type produces primarily IL-4, 5, 6, 10 and 13 which upregulates humoral or antibody mediated immunity via activation of B-cells and macrophages. We found that the expression of CD4 was decreased in transgenic cattle, and it stand to reason that the expression level of IL-2 and IFN-γ were decreased, in addition, the expression of IL-6 and IL-10 were decreased.

It is widely known that granulocyte-macrophage colony-stimulating-factor (GM-CSF) combination with cytokines to differentiate human peripheral blood monocytes into potent T cell-stimulatory cells and also has been involved in the spontaneous differentiation of human monocyte precursors into macrophages, by enhancing their survival [21,22]. GM-CSF is promote the differentiation of human blood monocytes into dendritic cell(DC) and that the number of DC achieved in the presence of GM-CSF alone, but not in combination with IL-4, correlates with the extent of GM-CSF receptor α expression [23]. The expression level of GM-CSFRα is down regulated in transgenic cattle.

A sequence in transgenic cattle which similar to ZAP-70 (Zeta-chain associated protein kinase 70 kDa) is down-regulation. ZAP-70, a cytoplasmic tyrosine kinase mainly expressed in T cells, and it plays a role in T-cell development and lymphocyte activation [24-27]. In rodents, it has been shown that stimulation through the TCR/CD3 complex is associated with reduced IL-2 production and subsequent proliferation [28]. Loss of ZAP-70 activation in response to TCR/CD3 receptor stimulation and subsequent suppression of IL-2 production [29].

Indoleamine 2, 3-dioxygenase (IDO2), which is the rate-limiting enzyme for tryptophan catabolism, may play a critical role in various inflammatory disorders [30]. IDO2 may be important to sustain immune escape, IDO2 seems to block the proliferation of alloreactive T lymphocytes through arrest in the G1 phase of the cell cycle [31-33]. The expression of IDO2 is increased in transgenic cattle.

Regulatory T cells (Treg) play an important role in maintain of homeostasis of the immune system capable of suppressing other immune responses in vitro and/or in vivo. The cattle CD4^+^CD25^high ^Foxp3^+ ^and CD4^+^CD25^low ^Foxp3^+^T cells do not function as Treg *ex vivo*. This indicates that the bovine immune system may be governed by different regulatory mechanisms as compared to rodents and humans. In the bovine immune system a role for monocytes has been suggested in the control of γδ T cell responses [34], probably mediated by IL-10 secretion [35]. The bovine Treg function appears to reside in the γδ T cell population, more precisely in the WC1.1^+ ^and the WC1.2^+ ^subpopulation, major populations present in blood of cattle [36], in this study the expression of WC1 in transgenic cattle is down regulation. Expression of LAG3 in human CD4^+^T cells and found that LAG3 identifies a discrete subset of CD4^+^CD25^high^Foxp3^+^T cells. CD4^+^CD25^high ^Foxp3^+^LAG3^+^T cells are functionally active cells that release the immunosuppressive cytokines IL-10 and TGF-β1 [37]. Nevertheless, the cattle CD4^+^CD25^high ^Foxp3^+ ^T cells do not function as Treg *ex vivo*[36], lower expression of LAG3 in transgenic cattle whether influence the immune should be further study.

Acetylserotonin O-methyltransferase (ASMT) is the enzyme involved in the last step of melatonin synthesis. Melatonin is a powerful antioxidant molecule involved in the protection of nuclear and mitochondrial DNA and in the regulation of circadian seasonal rhythms and immune function [38]. It is produced and secreted predominantly by the pineal gland. The proportions of ASMT-immunoreactive cells successively decreased in the pineocytoma [39]. Lower expression of ASMT in transgenic cattle may affect the melatonin synthesis and then influence the immune function.

Feeding purified EPA and DHA significantly reduced spleen lymphocyte proliferation, natural kill cell activity and PGE2 production in nonautoimmune prone mice [40]. It is consistent with the result that natural kill cells activity and the expression of prostaglandin E synthase are reduced in transgenic cattle.

### ω-3 fatty acids on inflammation and cancer

A large number of epidemiological studies and data in rodents implicate polyunsaturated fatty acid related with cancer particularly colon, breast, and prostate cancer [5,41,42]. They are complex diseases that are affected by both genetic and environmental factors. There have been advanced to explain that fatty acid composition effects on membrane ﬂuidity, cell signaling, hormone imbalance, and prostanoid synthesis [41-43]. Fatty acid effects on cell growth, differentiation, metabolism, and the production of eicosanoids, cytokines, and adhesion molecules are all likely to contribute to cancer cell growth.

The generation of proinﬂammatory cytokines, eicosanoids, and growth factor agonists and antagonists at the site of injury contributes to atherosclerosis [44]. To decrease the eicosanoid, cytokine, and adhesion molecule production is benefit to control the atherosclerosis process. The production of adhesion molecules (VCAM-1) from cultured endothelial cells is suppressed by ω-3 fatty acids [45]. Adhesive interactions between leucocytes and cellular or extracellular components of tissues are involved in inﬂammatory or immunological response mechanisms. Adhesion molecules direct the leucocyte-endothelium interactions, transendothelial migration of leucocytes and leucocyte trafﬁcking in general [46]. However, our date showed that expression of VCAM-1 in transgenic cattle is higher than wild-type cattle.

Eicosanoid is promote the tumourgenesis, which produced by COXs and LOXs to catalyze Amino acid or EPA. The antiproliferative effects of ω-3 fatty acids in cancers is inhibit the expression of cyclooxygenase 2 (COX2), at least partly [47]. However, the change of COX2 expression level is not detected in our data. To decrease the COX2 expression also can by regulate other genes indirectly, such as nitric oxide (NO). NO activates COX2 expression, the effect of DHA on COX2 could be to decrease NO indirectly. Narayanan et al had shown that treatment of human colon cancer cells with DHA downregulates inducible NO synthase [48]. NO also can cause cell damage in inflammation process, therefore, it is possible that sustained high levels of NO generated by iNOS can produce lead to tumor initiation and promotion various kinds of damage [49,50]. DHA could indeed induce cancer cell death via down-regulation of iNOS expression and/or by modulating sets of genes involved in apoptosis and differentiation [51]. Ntric oxide synthase was decreased in transgenic cattle in our study.

Arachidonic acid (AA) which is released from membrane phospholipids together with diacylglycerol during signal transduction activates the transcription factor or nuclear factor NF-kB, which then transmigrates into the cell nucleus and induces a number of the inﬂammatory genes, such as COX2, cytokines, and adhesion molecules. Inhibit of NF-kB signaling is contribute to the anti-inflammatory actions of DHA [52,53]. The expression NF-kB activation induced by arachidonic acid is decrease in transgenic cattle, in turn, down-regulates the transcription of genes regulating the inﬂammatory response (cytokines, chemokines, cell adhesion molecules). Berger A et al consistent with the result that hepatic NF-kB gene expression was downregulated by DHA [54].

Chondroitin sulfate proteoglycan 4 (CSPG4), also known as high Molecular Weight- Melanoma Associated Antigen, is a cell surface proteoglycan which has been recently shown to be expressed not only by melanoma cells, but also expressed by basal breast carcinoma, squamous carcinoma of the head and neck, mesothelioma, pancreatic carcinoma, some types of renal cell carcinoma, chordoma, and chondrosarcoma cells, however, its restricted distribution in normal tissues and cells [55]. So lower expression of CSPG4 in transgenic cattle may be a signal of reduce the risk of suffer from various types of cancer. Furthermore, there have other genes related with cancer showed in Table 1. There had reported that fat-1 mice with elevate ω-3 fatty acid is suppressed various tumorigenesis [56-59].

Huntingtin interacting protein-1 (HIP1) is known to be associated with the N-terminal domain of huntingtin. Overexpression of HIP1 induced cell death through caspase-3 activation in immortalized hippocampal neuroprogenitor cells [60], HIP1 overexpression was also found in several primary epithelial tumors including breast, ovarian, prostate, lung and colon, and its expression negatively correlated with survival in men with prostate cancers [61]. The expression of HIP1 in transgenic cattle is increased.

### ω-3 fatty acids on nervous development and neurologic disease

PUFAs have many important actions not only by themselves but also by giving raise to various biologically active compounds. PUFAs play a significant role in various diseases and especially in cardiovascular and neurological/psychiatric disorders [62]. Enrich the ω-3 fatty acids alter the composition of membranes. Alteration in the cellular architecture along with alterations in molecular composition of membranes might influence a wide range of brain functions: stabilization of axons and dendrites, cell shape, polarity, neural plasticity, vesicle formation and transport. Diet with high DHA slowed the progression of Alzheimer's disease (AD) in mice. Specifically, DHA cut the harmful brain plaques that mark the disease [62-64]. DHA protected against damage to the «synaptic» areas and enabled mice to perform better on memory tests [2]. The observation that ω-3 fatty acids, affect expression levels of a number of genes in brain opens the way toward understanding the role of these fatty acids in the function of central nervous tissue.

The proteins encode by protocadherin gamma subfamily most likely play a critical role in the establishment and function of specific cell-cell connections in the brain, such as PCDHGA9, PCDHGA8, PCDHGB4, PCDHGA6 [65], so higher expression of this genes may beneficial to brain development. KCNK10 is probably an important ion channel to involve in the neuroprotection by tuning the level of resting potential, reducing the brain cell excitability and release of stimulative neuro-transmitters. The expression of KCNK10 is increased when in the process of neuropathic pain and memory impaired [66]. The expression of KCNK10 in transgenic cattle is lower than wild-type. SEMA5B is involved in synapse elimination in hippocampal neurons. Overexpression of SEMA5B in hippocampal neurons results in a decrease in synapse number, however, depletion of endogenous SEMA5B using short hairpin RNA (shRNA) resulted in the exuberant formation and/or maintenance of synaptic connections, with a concomitant increase in the size of pre and postsynaptic densities [67], lower expression of this gene in transgenic cattle may increase the synaptic number and size in hippocampal neurons, and maybe increase the ability of learning and memory. Recent studies in fat-1 transgenic mice showed that increased brain DHA significantly enhances hippocampal neurogenesis as evidenced by an increase in the number of pro-liferating neurons and increased density of dendritic spines of CA1 pyramidal neurons in the hippocampus [68]. The study of fat-1 transgenic mice had demonstrated that higher level of ω-3 fatty acids is more effective in reaching the brain and achieving neuroprotection in an animal model of PD [69,70].

ω-3 fatty acids modulate brain growth and development, and neuronal differentiation. In addition, their ability to form an important constituent of neuronal cell membranes and involvement in memory formation and consolidation [71], explaining the beneficial action of EPA and DHA in the prevention and treatment of dementia and Alzheimer's disease [72,73]. However, different conclusion on DHA and EPA in neurological conditions had been present. Bate et al reported that pre-treatment with DHA or EPA significantly reduced the survival of cortical or cerebellar neurons, they noted that treatment with DHA or EPA reduced the free cholesterol content of neuronal membranes that increased the kinetics of incorporation [74,75]. These observations indicate that under some specific conditions ω-3 fatty acids (EPA and DHA) may actually accelerate neuronal loss in the terminal stages of prion or Alzheimer's diseases. Our dates not show adverse effect on neurological conditions.

Higher expression of Olfactory receptor, SEPTIN5 in transgenic cattle may strengthen the function of olfactory sense, visual sense respectively [76,77]. Suh M had demonstrated that fat-1 mice enriched highly ω-3 fatty acids in the retina lead to supernormal scotopic and photopic ERGs and increases in Muller cell reactivity and oxidative stress in photoreceptors [78].

### ω-3 fatty acids on fertility

PUFA composition of the cell membranes of the sperm and oocyte is important during fertilization [79]. Altering the PUFA sources in the diet resulted in concomitant changes in the ω-6 and ω-3 composition of sperm [80]. With regard to male fertility, PUFAs are essential by virtue of their ability to confer upon the sperm plasma membrane the fluidity it needs to achieve fertilization. Experiments on chickens have shown that feeding more PUFAs in the diet reduced the antioxidant status and quality of the semen [81].

Loss of the Calmegin (CLGN) lead to the production of sterile sperm that do not bind to the egg zone pellucida [82], so higher expression of CLNG in transgenic cattle might benefit to the spermatogenesis. EF-hand calcium binding domain 6 (EFCAB6) recruits histone-deacetylase complexes in order to repress transcription activity of androgen receptor (AR). The AR is a member of the nuclear receptor superfamily and plays a role as a ligand-dependent transcription factor. After a ligand binds to the AR, the AR is translocated into the nucleus and binds to the androgen-responsive element (ARE), on the androgen-activating gene that affects development, growth, and regulation of male reproductive functions [83,84]. Lower expression of EFCAB6 in transgenic cattle may lessen the suppression of AR and to express male-specific genes and the fertilization function of mature sperm.

Sex determining region Y-box 8 (SOX8) is expressed in the developing testis around the time of sex determination suggesting that it might play a role in regulating the expression of testis-specific genes [85], higher expression of SOX8 in transgenic cattle may receptor the sex determination.

## Conclusion

To study the effect of ω-3 fatty acids on various physiological processes and pathologic situations, traditional approach to modify tissue nutrient composition is by supplementing the experimental groups with different ω-3/ω-6 fatty acid ratios. Although this is an accepted mode of studying the effect, it is difficult to make all the dietary components identical. The inevitable differences between diets and their components, even if small they may be, may confound the study and contribute to inconsistencies or conflicting results observed. In these studies, fish oils or plant oils are used to provide the required ω-3/ω-6 fatty acids in generally. Since these fatty acids are derived from different sources and are likely to contain other bioactive compounds, however minor they might be, are likely to affect the study outcomes. It is necessary to develop a transgenic animal model more efficient converting ω-6 to ω-3 fatty acids, the results obtained in such model will be more reliable to interpret the function of ω-3 fatty acids.

Our data derived from the fat-1 transgenic cattle support the notion that a reduced ratio of ω-6/ω-3 fatty acids is favorable for normal cell function and may reduce the risk of certain diseases, such as cardiovascular disease, inflammatory disorders and cancer. Our result is generally consistent with studies using this model to address the effects of ω-3 fatty acids. However, we detected some gene expression are contrary to previous studies, for instance, the production of VCAM-1 from cultured endothelial cells is suppressed by n-3 PUFA, however, our date showed that expression of VCAM-1 in transgenic cattle is higher than wild-type cattle. In addition, the expression of WC1, ASMT and SOX8 were down-regulated and HIP1 expression was increased.

Due to the only three positive fat-1 transgenic cattle detected, the result of cDNA microarray is limited by the little number of samples. It is necessary to verify the conclusion using large-scale samples when transgenic cattle have generation.

## Competing interests

The authors declare that they have no competing interests.

## Authors' contributions

TG conceived and designed the study, carried out the experiments and wrote the manuscript. XF and XB helped to take samples. YW and FF participated in analysis data. YJ and HG participated in its design and coordination. All authors read and approved the final manuscript.
